# Wavelength- and Polarization-Multiplexed Dielectric Metasurface for On-Demand Generation and Continuous Tuning of Optical Fields

**DOI:** 10.3390/nano16201324

**Published:** 2026-10-20

**Authors:** Qi Zhang, Jiaqi Xing, Haoze Pan, Shun Zhou, Qi Xu, Nana Wang, Changda Zhou

**Affiliations:** 1 School of Medical Information Engineering, Jining Medical University, Jining 272067, China; 2 State Key Laboratory of Optoelectronic Materials and Technologies, School of Physics, Sun Yat-sen University, Guangzhou 510275, China; 3 School of Criminal Science and Technology, Shandong Police College, Jinan 250200, China

**Keywords:** dielectric metasurfaces, wavelength-polarization multiplexing, perfect vortex beams, wavefront control, on-demand optical-field generation, continuous modal control

## Abstract

Despite progress in optical multiplexing, integrating discrete channel switching with continuous control of distinct optical fields in a single metasurface remains challenging. Here, we numerically demonstrate a dielectric metasurface that enables wavelength- and polarization-selective generation of focal spots and perfect vortex beams (PVBs), with their relative contributions continuously tuned via the incident ellipticity without reconfiguring the structure. This functionality is enabled by combining wavelength-selective Si nanopillars with Pancharatnam–Berry geometric-phase encoding, which decouples wavelength selectivity from phase control. Under circularly polarized illumination, focal spots and PVBs are selectively generated at both 532 nm and 632.8 nm. The generated PVBs exhibit a common ring radius with wavelength-dependent topological charges of *l* = +4 at 532 nm and *l* = −2 at 632.8 nm, and their modal purities reach 79.7% and 97.7%, respectively. Under horizontal linearly polarized light, both circular-polarization channels are simultaneously excited to produce a hybrid field composed of a focal spot and a PVB. As the ellipticity increases, the ratio of PVB to focal spot intensity rises continuously, in good agreement with the analytical modal. The flexibility of the proposed approach is further demonstrated by off-axis focusing, non-uniform phase PVB generation, and control of the PVB ring radius. This work provides a reconfiguration-free platform that combines wavelength- and polarization-selective switching with continuous control of relative modal contributions, offering potential applications in optical trapping and mode-division multiplexing.

## 1. Introduction

Precise manipulation of optical fields, including the amplitude, phase, polarization, wavelength and orbital angular momentum (OAM), has attracted substantial research interest in modern photonics [1,2]. Such advanced optical-field engineering has enabled numerous applications ranging from super-resolution imaging and sensing to information processing and optical manipulation [3,4,5]. Conventional optical components, such as lenses and waveplates, rely on propagation through bulk materials to achieve phase retardation and polarization control. However, the relatively bulky configurations limit their compactness and functional integration [1]. Metasurfaces, consisting of subwavelength structures arranged on a planar interface, provide a powerful platform for replacing conventional optical components and enabling flexible control of the amplitude, phase, polarization, and wavefront of light [6,7]. In particular, dielectric metasurfaces have attracted considerable attention due to their low optical loss and efficient wavefront manipulation [8,9,10,11]. Silicon (Si) is an attractive material for dielectric metasurfaces, particularly in the visible and near-infrared regions, owing to its high refractive index and compatibility with established nanofabrication technologies [12,13]. Anisotropic nanostructures, such as rectangular Si nanopillars, can be designed to provide polarization-dependent optical responses [9,14]. By rotating such anisotropic nanostructures, Pancharatnam–Berry (P-B) geometric phase can be induced to the cross-polarization component, enabling full 2π phase modulation [15,16]. This combination provides additional degrees of freedom for encoding multiple optical functions within a single metasurface. Structured-light generation provides an important avenue for advanced optical-field manipulation using metasurfaces [17,18,19,20]. Among the diverse structured light, optical vortex beams carrying OAM have attracted considerable interest due to their helical phase fronts, phase singularities and unique light–matter interaction properties [21]. Perfect vortex beams (PVBs) are particularly attractive because their annular intensity distribution can maintain a constant ring radius independent of the topological charge [18,19]. This unique property facilitates spatial mode matching among different OAM channels, making PVBs particularly attractive for nonlinear frequency conversion and optical communication [22].

In recent years, driven by the increasing demand of high-information capacity and functional integration, the utilization of multiple optical degrees of freedom for multiplexed manipulation has attracted increasing attention [23,24,25,26,27]. Among the various approaches, polarization and wavelength are particularly attractive because they can be directly controlled by the incident light without reconfiguring the metasurface. Polarization multiplexing enables different wavefronts to be selectively manipulated according to the polarization state of incident light [23,24]. Meanwhile, wavelength-dependent responses of meta-atoms can be exploited to distinguish different spectral channels and realize wavelength-selective optical functions [26]. Beyond single-freedom modulation, the combination of wavelength and polarization has further expanded the functional capacities of metasurfaces, enabling multiple optical responses to be addressed through different wavelength-polarization combinations [28,29,30,31,32]. Recent studies have demonstrated high-capacity wavelength-polarization multiplexing with multiple independent channels and multichannel wavefront manipulation [29,31]. In addition to discrete channel selection, continuous control enabled by polarization or wavelength has also been explored, extending multiplexing toward continuous parameter mapping [32]. These developments indicate that wavelength and polarization have evolved from simple switching parameters into powerful degrees of freedom for multidimensional optical-field control. Nevertheless, existing multiplexed metasurfaces mainly focus on discrete channel switching or continuous modulation of individual or similar optical fields. The simultaneous generation of two distinct optical fields and continuous control of their relative contributions within a single metasurface has rarely been reported.

In this work, we propose and numerically demonstrate a multifunctional dielectric metasurface that achieves simultaneous wavelength- and polarization-selective wavefront modulation for on-demand optical-field generation. The metasurface, composed of rectangular Si nanopillars, is optimized to exhibit both wavelength-selective responses and polarization-dependent P-B phase control. The two typical visible wavelengths 532 nm and 632.8 nm are chosen because they correspond to common solid-state and He-Ne laser sources, and their approximately 100 nm spectral separation is sufficient to distinguish the two wavelength channels for wavelength-selective optical-field generation. By switching the handedness of incident circularly polarized light, the desired optical field can be selectively generated at the designed wavelengths, enabling focal spot and PVB generation. The generated PVBs exhibit wavelength-dependent topological charges of *l* = +4 at 532 nm and *l* = −2 at 632.8 nm. These values are selected as representative examples to show that PVBs with different topological charges and opposite signs can share a common ring radius, while the proposed approach can be extended to other topological charges according to specific design requirements. In addition, both PVBs exhibit high modal purities. Under horizontal linearly polarized illumination, both channels are simultaneously excited to produce composite optical fields. Crucially, by controlling the ellipticity of the incident light, the relative contributions between the focal spot and the PVB can be continuously tuned without metasurface reconfiguration. The flexibility of our design is further validated by demonstrating off-axis focal spots, non-uniform phase PVBs, and PVBs with varied ring radii. The key contribution of the proposed approach is to combine wavelength-selective and polarization-dependent optical-field generation with continuous tuning of the relative contributions of two distinct optical modes within a single metasurface. Rather than introducing a new polarization-weighting mechanism, this approach exploits the intrinsic ellipticity-dependent weighting of geometric-phase-encoded channels to achieve reconfiguration-free modal control. These results establish a compact, reconfiguration-free platform for multifunctional structured-light generation, enabling flexible mode control in photonic systems. Such capabilities are promising for applications in optical trapping and manipulation [1,5], mode-division multiplexing and structured-light communication [2,21,27], nonlinear frequency conversion [22], and integrated multifunctional photonic devices [29,30,31,32].

## 2. Metasurface Design and Operating Principle

### 2.1. Wavelength- and Polarization-Multiplexed Metasurface Design

**Figure 1:**
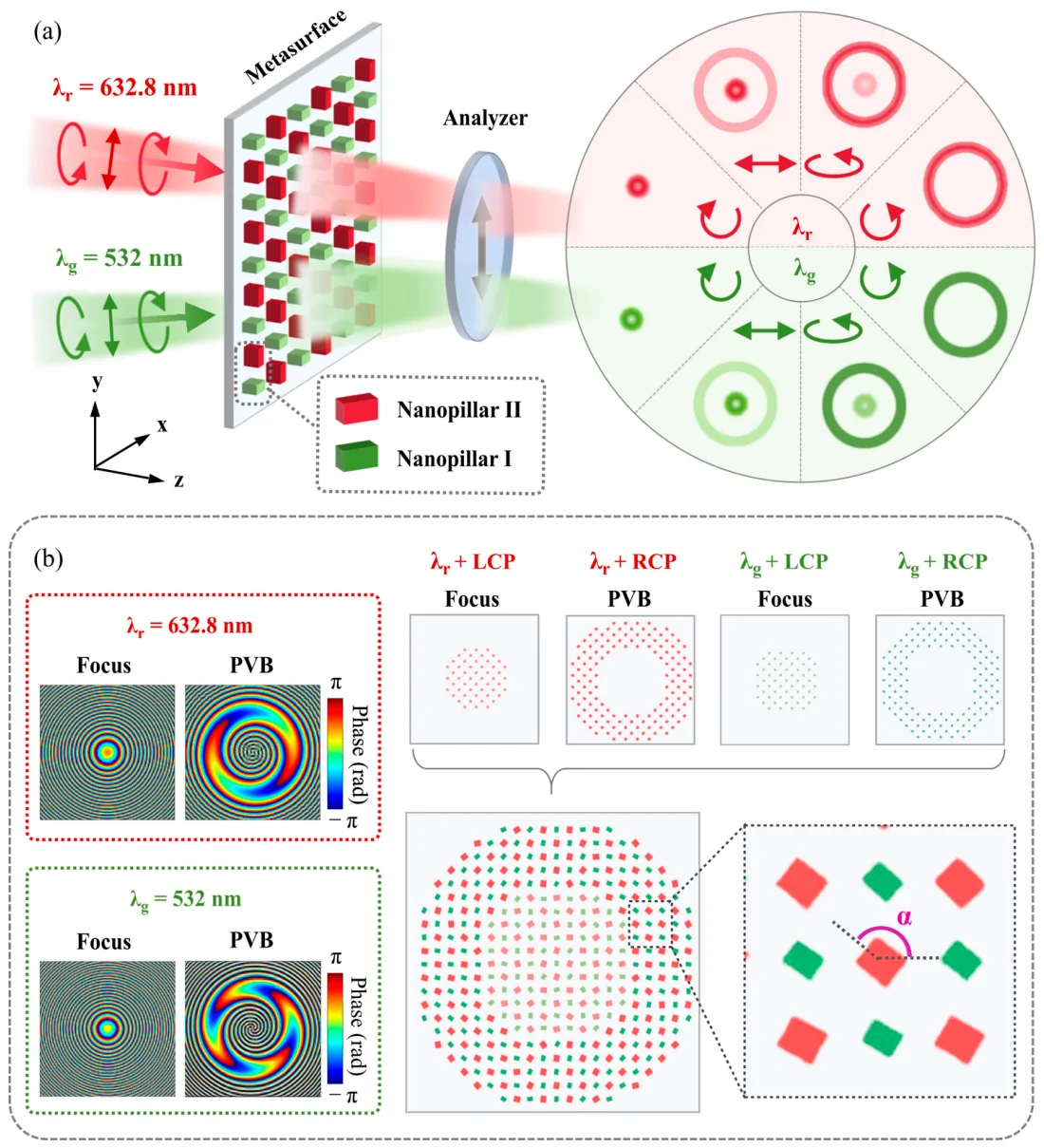
Design concept and holographic phase encoding of the wavelength- and polarization-multiplexed metasurface. (**a**) Schematic of the device functionality. The metasurface enables on-demand generation of focal spots, perfect vortex beams (PVBs), or a hybrid field composed by a focal spot and a PVB by controlling the wavelength and polarization state of the incident light. (**b**) Designed holographic phase distributions and corresponding nanopillar arrangements for the four operating channels, together with the top view of the integrated metasurface. The magnified view illustrates the in-plane rotation angle α of a representative nanopillar.

The main functionality of the proposed metasurface is illustrated in Figure 1a. By controlling the wavelength and polarization state of the incident light, the metasurface enables on-demand generation of focal spots, PVBs, and their hybrid field. Under circularly polarized illumination, the output optical field depends on both the incident wavelength and polarization handedness, selectively generating focal spots or PVBs. Under linearly or elliptically polarized light, the left-handed circularly polarized (LCP) and right-handed circularly polarized (RCP) channels are simultaneously excited, resulting in a hybrid field composed of a focal spot and a PVB. In particular, the ellipticity of the incident polarized light provides continuous control over the relative contributions of the focal spot and the PVB.

This multifunctional behavior is realized by a carefully designed spatial arrangement of rectangular Si nanopillars, and the design principle is illustrated in Figure 1b. Based on Fresnel holography technology, the required phase distribution on the metasurface is obtained by applying inverse Fresnel diffraction to the target field at the focal plane [33]. For the focusing channels, the complex amplitude of the target field at the focal plane can be expressed as

(1) $$E_{0}^{\mathrm{focus}}\left(x_{0},y_{0}\right)=A\exp\left[-\frac{{\left(x_{0}-x_{f}\right)}^{2}+{\left(y_{0}-y_{f}\right)}^{2}}{w_{0}^{2}}\right],$$

where *A* and *w*_0_ denote the amplitude and waist of the focal spots, respectively. (*x_f_*, *y_f_*) represents the position coordinates of the focal spot at the target plane. For the PVB channels, the target field is defined as an annular beam characterized by a topological charge *l* and ring radius *r*_0_, which can be written as

(2) $$E_{0}^{\mathrm{PVB}}\left(x_{0},y_{0}\right)=A\exp\left[-\frac{{\left(\sqrt{{x_{0}}^{2}+{y_{0}}^{2}}-r_{0}\right)}^{2}}{w_{0}^{2}}\right]e^{il\theta },$$

where *θ* = arctan(*y_0_/x_0_*) is the azimuthal angle. Then, the required complex amplitude at the metasurface for each channel is calculated by the inverse Fresnel diffraction as

(3) $$E_{meta}\left(x,y\right)=\frac{e^{ikf}}{ikf}\iint E_{0}(x_{0},y_{0})\exp\left\{\frac{ik}{2f}\left[{\left(x-x_{0}\right)}^{2}+{\left(y-x_{0}\right)}^{2}\right]\right\}dx_{0}dy_{0},$$

where *f* is the distance from the target plane to the metasurface, *k* = 2π/*λ* is the wavenumber and *λ* is the wavelength. The phase of *E_meta_*(*x*,*y*), denoted by *Φ*(*x*, *y*), defines the required holographic phase distribution for the corresponding channel. The calculated holographic phase distributions for the four working channels are shown in Figure 1b.

To integrate the four channels into a single metasurface, we employ a spatial multiplexing method. The inner and outer regions respectively encode the focal spot and the PVB phase channels for both wavelengths under LCP and RCP illumination. In each spatial region, the two wavelength channels are interleaved in a checkerboard pattern, ensuring an approximately uniform spatial distribution of the wavelength-selective nanopillars across the metasurface. Therefore, the phase distribution encoded on the metasurface is given by

(4) $$\Phi_{meta}\left(x,y\right)=\left\{\begin{array}{c} \begin{array}{cc} \Phi_{\mathrm{focus}}\left(x,y\right), & \sqrt{x^{2}+y^{2}}\leq R_{1} \end{array}\\ \begin{array}{cc} \Phi_{\mathrm{PVB}}\left(x,y\right), & R_{1}<\sqrt{x^{2}+y^{2}}\leq R_{2} \end{array} \end{array}\right.,$$

where *R*_1_ = 5 μm and *R*_2_ = 15 μm define the boundaries of the inner and outer regions, respectively.

The holographic phase distributions are physically encoded into the metasurface using the P-B geometric-phase principle [15,16]. The optimized rectangular Si nanopillar can act as a half-wave plate, with its fast and slow axes along length and width directions, respectively. For a nanopillar rotated counterclockwise by an angle *α* with respect to the *x*-axis, the Jones matrix of the nanopillar can be written as

(5) $$J(\alpha )=R^{-1}(\alpha )\left(\begin{array}{cc} t_{L} & 0\\ 0 & t_{W} \end{array}\right)R(\alpha ).$$

where *t_L_* and *t_W_* are the complex transmission coefficients along the length and width directions, respectively. The rotation matrix is expressed as

(6) $$R(\alpha )=\left(\begin{array}{cc} \cos\alpha & \sin\alpha \\ -\sin\alpha & \cos\alpha \end{array}\right).$$

For an incident circularly polarized light **E**_in_ = **e**_σ_ = (1, *iσ*)^T^, where *σ =* +1 corresponding to LCP light and *σ =* −1 corresponding to RCP light, the transmitted field is given by

(7) $$E_{\mathrm{out}}=J(\alpha )E_{\mathrm{in}}=\frac{t_{L}+t_{W}}{2}e_{\sigma }+\frac{t_{L}-t_{W}}{2}e^{i2\sigma \alpha }e_{-\sigma }.$$

For the cross-polarized component, the transmitted field can be expressed as

(8) $$E_{\mathrm{cross}}\propto \frac{t_{L}-t_{W}}{2}e^{i2\sigma \alpha }E_{\mathrm{in}}.$$

where the wavelength-dependent transmission coefficients *t_L_* and *t_W_* determine the conversion amplitude, while 2*σα* represents the P-B geometric phase. Therefore, wavelength selectivity is primarily governed by the dispersive response of the nanopillar geometry, whereas the phase profile is encoded through the in-plane rotation angle *α*. To encode a target holographic phase Φ(*x,y*) for a specific polarization channel, the rotation angle of the nanopillars on the metasurface can be determined as

(9) $$\alpha_{meta}\left(x,y\right)=\frac{\Phi_{meta}\left(x,y\right)}{2}.$$

This simple relationship between the rotation angle and the desired phase allows the four independent phase distributions shown in Figure 1b to be simultaneously encoded into the inner and outer regions of the metasurface.

### 2.2. Design and Optical Response of Wavelength-Selective Nanopillars

**Figure 2:**
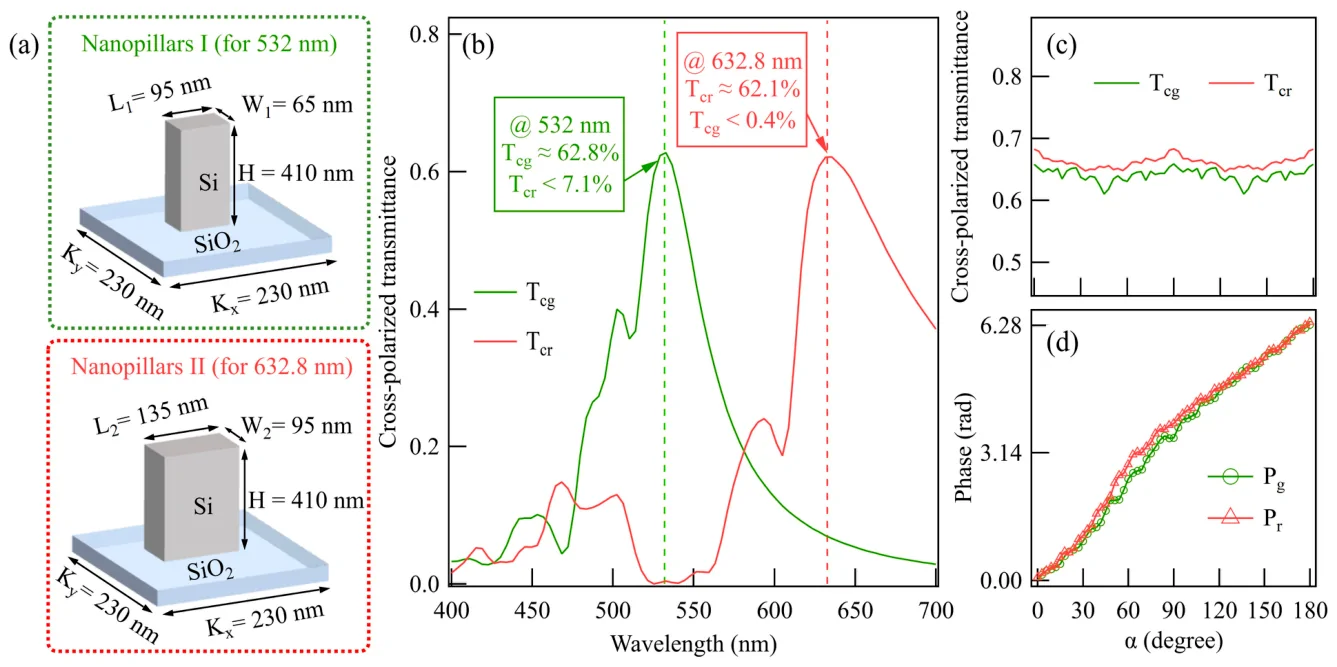
Design and optical response of the wavelength-selective dielectric nanopillars. (**a**) Schematic diagrams and geometric parameters of nanopillar I and nanopillar II designed for operation at 532 and 632.8 nm, respectively. (**b**) Cross-polarized transmittance spectra of two nanopillars, exhibiting high cross-polarized transmittance at their respective design wavelengths and strongly suppressed transmittance at the other wavelength. (**c**) Cross-polarized transmittance as a function of the in-plane rotation angle α at the corresponding operating wavelengths. (**d**) Phase response as a function of the rotation angle α, showing the required 2π phase coverage for geometric-phase encoding.

Figure 2a shows the rectangular Si nanopillars deposited on the SiO_2_ substrate. Si is chosen due to its high refractive index [12], and the SiO_2_ substrate provides high transmittance in the visible wavelength range [34]. The Si nanopillar possesses a fixed height of *H* = 410 nm, and its period is designed as *K*_x_ = *K*_y_ = 230 nm to provide enough spatial sampling of the target optical wavefront. It should be pointed out that the period is not an intrinsically required value, but a balanced compromise among subwavelength operation at both wavelengths, cross-polarized transmittances at the two operating wavelengths, and sufficient as well as roughly symmetric phase sampling for the nested focusing and PVB regions. A larger period still preserves the wavelength selectivity and the P-B phase response, and remains viable when the spectral selectivity and channel uniformity requirements are less demanding. The simulation results are obtained using the finite-difference time-domain (FDTD) method. The plane wave with left-circular polarization propagating along the *z*-direction is normally incident on the nanopillars. The periodic boundary conditions are applied along the *x*- and *y*-directions, while perfectly matched layers (PML) are applied along the *z*-direction to absorb the outgoing radiations. The cross-polarized transmittance *T*_c_, defined as the ratio of the transmitted RCP light power *P_cross_* to the incident LCP light power *P_in_*, is used to evaluate the performance of the nanopillars. The length *L* and width *W* of the nanopillar are optimized by analyzing the spectral response of *T*_c_ to obtain the desired wavelength-selective function. The detailed parameter optimization procedure and sweep results are provided in Appendix A. Figure 2b shows the cross-polarized transmittance spectra of two optimized nanopillars with different *L* and *W*. Nanopillar I with *L*_1_ = 95 nm and *W*_1_ = 65 nm exhibits a resonance peak at 532 nm, as shown by the green curve *T*_cg_. The red curve *T*_rg_ is the cross-polarized transmittance spectra of nanopillar II with *L*_2_ = 135 nm and *W*_2_ = 95 nm, which displays a resonance peak at 632.8 nm. Both nanopillars achieve high cross-polarization conversions at their designed wavelength, reaching *T*_cg_ = 62.8% at 532 nm and *T*_cr_ = 62.1% at 632.8 nm. In contrast, the corresponding *T*_c_ at the other wavelength is strongly suppressed to 0.4% and 7.1%, respectively. The wavelength-selective ability originates from the anisotropic optical properties of the rectangular Si nanopillar. *T_c_* = *P*_cross_/*P*_in_ is also determined by both the total transmittance *T = P*_out_/*P*_in_ and the polarization-conversion ratio *R_P_ = P*_cross_/*P*_out_, where *P*_out_ is the total transmitted power, i.e., *T_c_* = *T*·*R_p_*. At the design wavelength, the nanopillar exhibits high *T*, as well the phase retardation and amplitudes between its long and short axes matching half-waveplate condition, giving a high *R_P_*; both factors lead to a high *T_c_*. At the non-target wavelength, *T_c_* is suppressed by a reduction in *T*, *R_P_*, or both. The wavelength selectivity of the two nanopillars is further quantified by the crosstalk ratio, defined as

(10) $$C_{Τ}=\frac{T_{c\lambda_{other}}}{T_{c\lambda_{design}}}\times 100\%.$$

The corresponding crosstalk ratios are *C*_T,I_ = 11.3% and *C*_T,II_ = 0.6% for nanopillars I and II, respectively. Despite the difference in crosstalk levels, the results demonstrate pronounced wavelength selectivity between the two channels. In principle, the crosstalk ratio can approach zero if *T_c_* at the non-target wavelength vanishes while that at the target wavelength remains finite. However, in practice, the achievable limit is set by the residual off-resonance polarization conversion and the spectral overlap between the two channels. The wavelength-selective ability of the Si nanopillars depends on the geometrical sizes, and dimensional deviations may affect the conversion efficiency and inter-channel crosstalk. To evaluate the dependence, an additional tolerance analysis of nanopillar I and II by independently varying their length and width around the designed values are performed in Appendix B. These analyses identify the dimensional parameters most affecting the crosstalk ratio and provides a unit-cell-level assessment of dimensional tolerance as guidance for dimensional control in nanofabrication.

Figure 2c,d display the simulated cross-polarized transmittance and phase shift as functions of the nanopillar rotation angle α, respectively. As the nanopillars are rotated from 0° to 180°, the phase covers a full 2π range while maintaining a relatively constant *T*_c_, which consists with the P-B phase principle. Therefore, the nanopillar dimensions and rotation angle can be independently optimized for wavelength-selective polarization conversion and phase control, respectively. The unit-cell libraries can thus be constructed to realize required phase profiles for different wavelengths and polarization channels. Importantly, the wavelength selectivity and phase control originate from different physical mechanisms. The wavelength-dependent conversion is determined by the nanopillar dimensions through their dispersive optical response, whereas the P-B phase is controlled by the in-plane rotation angle α. Thus, the nanopillar dimensions and rotation angle provide two independent degrees of freedom for separately engineering wavelength-selective polarization conversion and geometric-phase encoding.

## 3. Simulation Results and Discussions

### 3.1. On-Demand Optical-Field Generation

**Figure 3:**
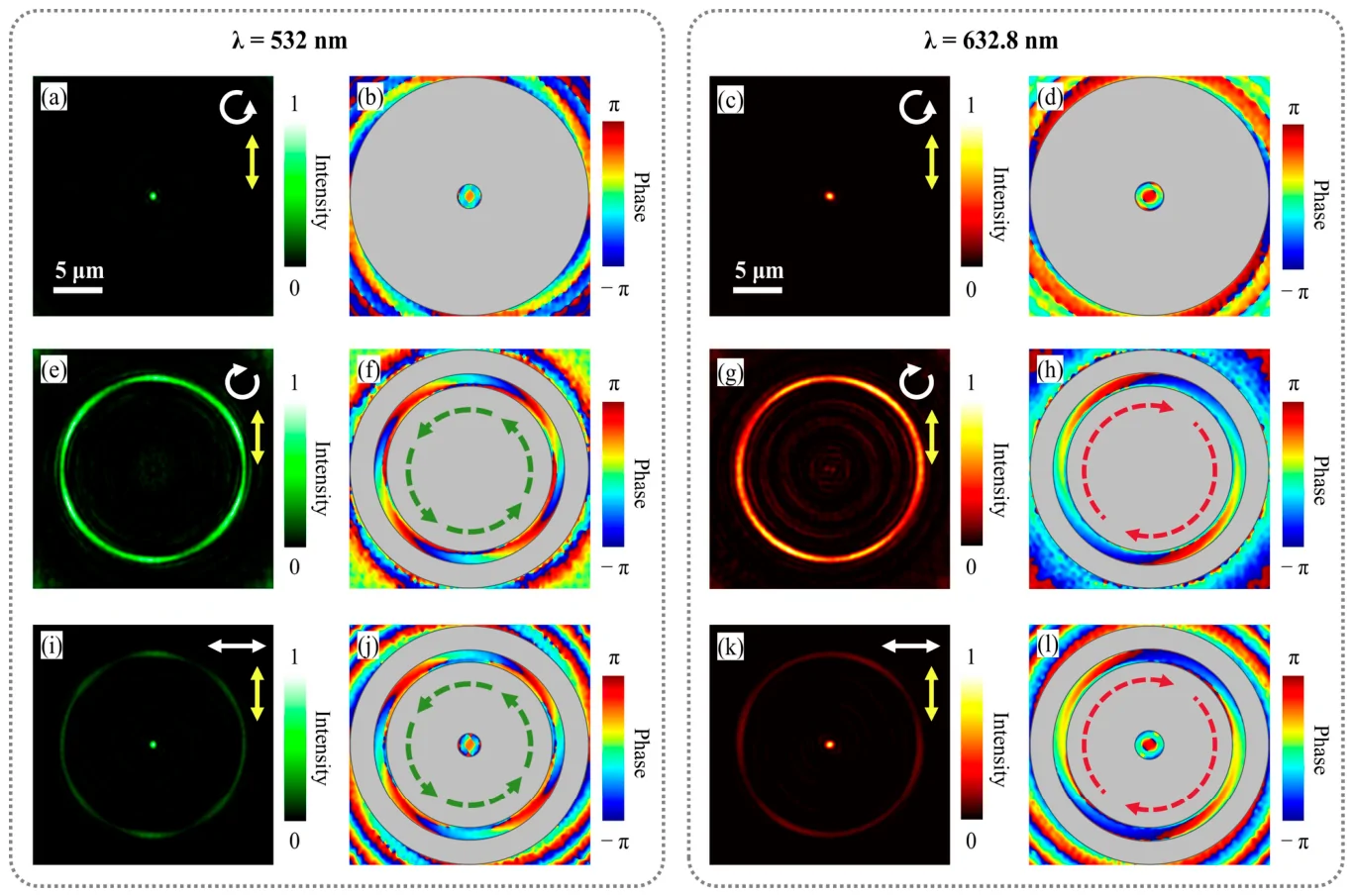
Simulated normalized intensity and phase distributions of *y*-component of the transmitted field under different polarization conditions. (**a**,**b**) Focal spots generated under left-circularly polarized (LCP) illumination at 532 nm. (**c**,**d**) Focal spots generated under LCP illumination at 632.8 nm. (**e**,**f**) PVB with topological charge *l* = +4 generated under right-handed circularly polarized (RCP) illumination at 532 nm. (**g**,**h**) PVB with topological charge *l* = −2 generated under RCP illumination at 632.8 nm. (**i**,**j**) Hybrid fields composed of a focal spot and a PVB generated under horizontal linearly polarized (HLP) illumination at 532 nm. (**k**,**l**) Hybrid fields composed of a focal spot and a PVB generated under HLP illumination at 632.8 nm.

Based on the design principle described in Section 2, a wavelength- and polarization-multiplexed metasurface is designed to enable discrete selection of distinct optical modes through wavelength and polarization control. At the target focal plane of *f* = 7 μm, LCP illumination generates focal spots at the origin, whereas RCP illumination generates PVBs. The PVB channels are designed with a common ring radius of *r*_0_ = 9 μm and topological charges of *l* = +4 at 532 nm and *l* = −2 at 632.8 nm. To validate the multifunctional performance of the designed metasurface, we numerically simulated the transmitted optical fields under various illumination conditions using the FDTD method. The simulation region is set to 30 μm × 30 μm in the transverse plane. Automatic mesh refinement and PML boundary conditions are used in the simulations. To capture the generated focal spots and PVBs, the observation plane is positioned at *f* = 7.3 μm, which is slightly larger than the designed focal length. The simulated *y*-components of the transmitted fields are shown in Figure 3, and all intensity distributions are normalized to their respective maximum values.

Figure 3a–d display the transmitted optical fields under LCP illumination, where the white and yellow arrows represent the incident polarization and the selected polarization component of the transmitted field, respectively. At both operating wavelengths of 532 nm and 632.8 nm, focal spots are observed at the center of the target plane. The corresponding phase distributions are uniform across the focal spot region, indicating that the transmitted wavefronts are coherently convergent at the focal spots. The full width at half maximum (FWHM) of the focal spots are 0.547 μm and 0.701 μm for the wavelengths of 532 nm and 632.8 nm, respectively, corresponding to effective numerical apertures (NA) of 0.496 and 0.46. Both values are lower than the designed NA of 0.582. Under RCP illumination, the metasurface switches its functionality from focusing to PVBs generation. The transmitted fields are shown in Figure 3e–h. At 532 nm, an annular intensity distribution with a ring radius of *r*_0_ = 9 μm is observed in Figure 3e. The corresponding phase distribution in Figure 3f shows a phase accumulation of 8π over one anticlockwise loop, indicating a topological charge of *l* = +4. As shown in Figure 3g,h, the transmitted field maintains the same annular radius of 9 μm at 632.8 nm. The phase distribution exhibits a 4π phase winding in the clockwise direction, demonstrating a topological charge of *l* = −2. Both PVB channels maintain the same designed ring radius despite having different topological charges. To evaluate the purity of the targeted topological charge modes and exclude the influence of the residual zero topological charge mode background light, the relative mode purity is defined as

(11) $$\eta_{l}=\frac{P_{l}}{\sum_{l^{\prime }\neq 0}P_{l^{\prime }}}.$$

where *P_l_* is the power of the target topological charge mode, and the denominator represents the total power of all nonzero topological charge modes, and the topological charge spectrum is analyzed around the ring radius with an annular region of 7 μm ≤ *r* ≤ 11 μm. The relative mode purities are 79.7% for *l* = +4 at 532 nm and 97.7% for *l* = −2 at 632.8 nm, confirming the high quality of the generated PVBs at both wavelengths. The total transmission efficiencies *T* of the complete metasurface are 38.9% at 532 nm and 53.3% at 632.8 nm. The higher total transmission efficiency at 632.8 nm can be attributed to the residual *T_c_* of Nanopillar I at 632.8 nm, which is much larger than that of Nanopillar II at 532 nm. To further evaluate the performance of the metasurface, the absolute efficiency of each channel is defined as the ratio of the power in the target optical-field region at the focal plane to the total incident power. Here, the target optical-field region refers to the focal spot area for the focusing channels and the annular region for the PVB channels. This absolute efficiency also reflects the conversion efficiency of each channel, since the target optical field is generated by the cross-polarized component. For the focal spot channels, the absolute efficiencies are 1.7% at 532 nm and 4.3% at 632.8 nm. The PVB channels exhibit absolute efficiencies of 19.3% at 532 nm and 25.9% at 632.8 nm. Due to the focusing channels occupying only about 11% of the total aperture area, their absolute efficiencies are lower than those of the PVB channels. Because the two wavelength-selective sets of nanopillars are interleaved, *T_c_* of each set at the target and non-target wavelengths cannot be individually extracted in the full metasurface. Therefore, we estimate the full metasurface wavelength crosstalk from the ratio of the non-target mode purity to the target mode purity of the generated PVBs. In the ideal case, the metasurface generates PVBs that exhibit topological charge *l* = +4 at 532 nm and *l* = −2 at 632.8 nm. Any residual *l* = −2 component at 532 nm originates from the 632.8 nm channel, and vice versa. At 532 nm, the non-target *l* = −2 mode purity is 4.6%, giving a crosstalk ratio of 4.6%/79.7% ≈ 5.77%. At 632.8 nm, the non-target *l* = +4 mode purity is 0.7%, giving a crosstalk ratio of 0.7%/97.7% ≈ 0.72%. Under horizontal linearly polarized (HLP) illumination, which can be expressed as an equal-amplitude coherent superposition of LCP and RCP components, the focusing and PVB channels can be simultaneously excited, as shown in Figure 3i–l. At 532 nm, the transmitted field composed of a central focal spot and a PVB is observed in Figure 3i,j. The hybrid field results from the coherent superposition of the focal spot generated by the LCP component and the PVB field generated by the RCP component. A similar hybrid optical field is observed at 632.8 nm, as shown in Figure 3k,l, confirming that the metasurface maintains the polarization-multiplexing capability at both wavelengths.

These results demonstrate that the metasurface enables on-demand generation of focal spots and PVBs with wavelength-dependent topological charges by switching the handedness and wavelength of incident circularly polarized light, while composite fields can be generated under HLP light. The demonstrated functionality provides a promising route toward multifunctional optical-field generation. It should be noted that the beam quality of the proposed metasurface is affected by the finite aperture and the spatial multiplexing scheme. The checkerboard interleaving reduces the effective sampling area available to each channel, and the abrupt transition between the phase profiles encoded in the focusing and PVB regions may lead to additional scattering and background light. These factors may limit the optical power delivered to the desired fields and affect their quality. Consistent with these limitations, the effective NAs of focal spots are smaller than the designed value. The present design represents a trade-off between beam quality and the realization of four channels in a single-layer, reconfiguration-free metasurface. Although the performance penalties associated with the finite aperture and spatial multiplexing cannot be completely eliminated in the current design, they may be mitigated by increasing the aperture size and optimizing the spatial interleaving strategy.

### 3.2. Continuous Modal Weighting via Polarization Ellipticity

In addition to discrete switching, the relative contributions of the two optical modes can be continuously controlled by varying the ellipticity of the incident light.

**Figure 4:**
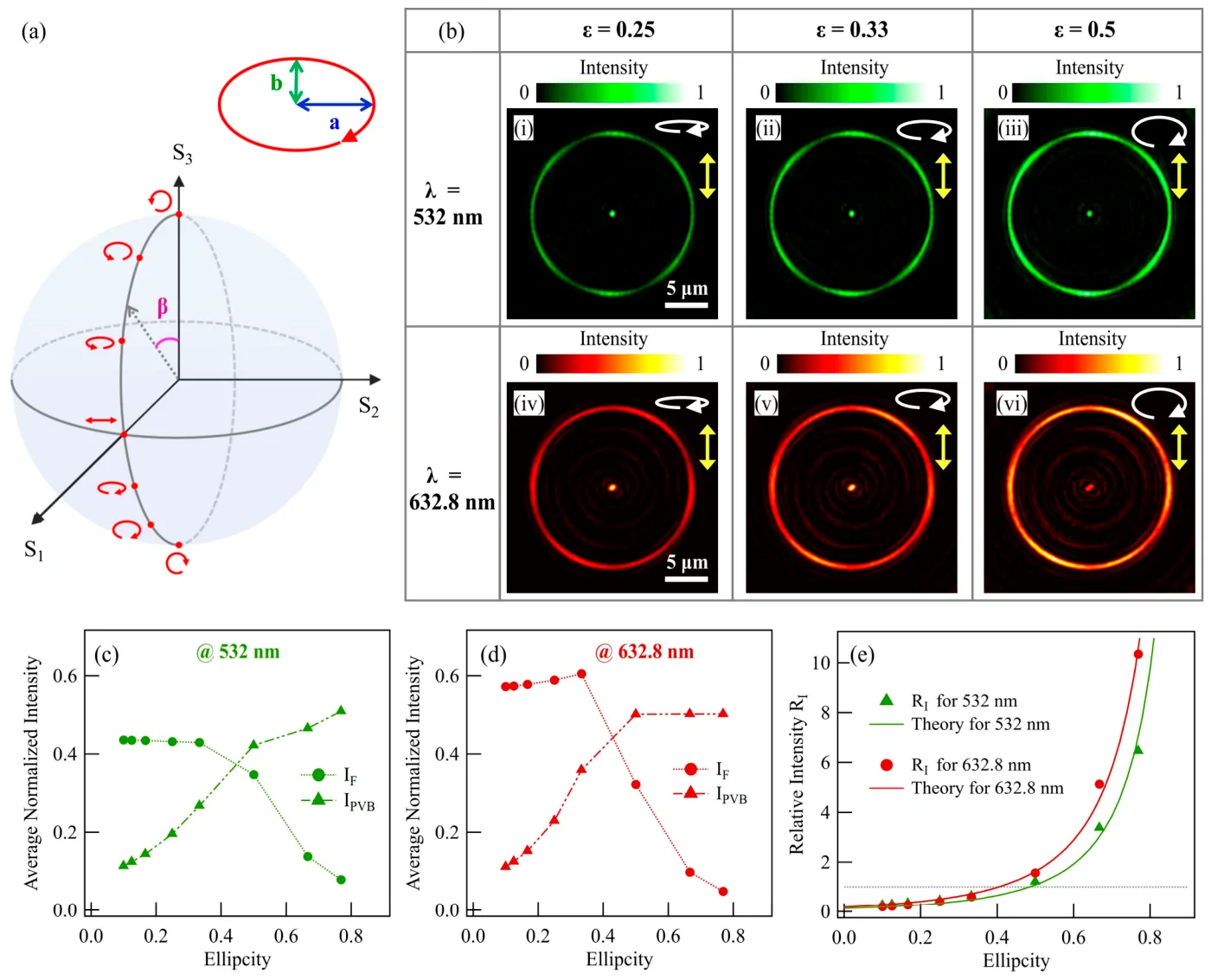
Continuous intensity tuning between the focal spot and PVB by controlling the ellipticity. (**a**) Polarization-state evolution from RCP to LCP on the Poincaré sphere. The inset illustrates the major axis *a* and minor axis *b* of the incident right-handed elliptically polarized light (REP). (**b**) Normalized intensity distributions under REP illumination with ellipticities of *ε* = 0.25, 0.33, and 0.5 at (i)–(iii) λ = 532 nm and (iv)–(vi) λ = 632.8 nm. Average normalized intensities of the focal spot *I*_F_ and PVB *I*_PVB_ as a function of the ellipticity at (**c**) 532 nm and (**d**) 632.8 nm, respectively. (**e**) PVB to focal spot relative intensity *R*_I_ as a function of the ellipticity.

This continuous modal control depends on the relative amplitudes of the two orthogonal circularly polarized components of the incident light, which correspond to two geometric-phase-encoded channels with distinct functions. As the incident ellipticity varies, the relative amplitudes of the LCP and RCP components change, which modifies the relative excitation strengths of the focal spot and PVB channels. This is an intrinsic property of P-B phase rather than a new physical mechanism. In the present design, we exploit this inherent property to achieve continuous control of the relative contributions of two distinct optical modes within a wavelength-selective, polarization-multiplexed metasurface. Here, we consider the polarization evolution from RCP toward HLP through right-handed elliptically polarized (REP) light along the lower hemisphere of the Poincaré sphere, as illustrated in Figure 4a. The major and minor axes are oriented horizontally and vertically, respectively, with their corresponding semi-axis amplitudes denoted by *a* and *b,* where *a* > *b*. The incident REP light can be decomposed into LCP and RCP components with amplitudes *A*_L_ and *A*_R_, respectively, and their amplitude ratio is given by

(12) $$\frac{A_{R}}{A_{L}}=\frac{1+\varepsilon }{1-\varepsilon },$$

where *ε = b/a* denotes the ellipticity of the REP light. Here, 0 < *ε* < 1, since *b* < *a* for the REP light considered. Therefore, the relative excitation strength of the PVB and focal spot channels are determined by the amplitude ratio between the RCP and LCP components. Accordingly, the relative intensity between the generated PVB and focal spot can be approximately described as

(13) $$R_{I}\approx C\frac{{\left(1+\varepsilon \right)}^{2}}{{\left(1-\varepsilon \right)}^{2}}.$$

where *C* is a proportionality factor that accounts for the different effective responses of the focal spot and PVB channels. When *R_I_* > 1, the PVB is stronger than the focal spot. While *R_I_* = 1, the two modes contribute equally. When *R_I_* < 1, the focal spot dominates the generated optical field. To demonstrate this continuous tuning capability, the metasurface is illuminated by the REP light with ellipticities from 0.1 to 0.77.

Figure 4b(i–iii) present the normalized intensity distributions at 532 nm under three typical conditions. The PVB rings are weaker than the focal spots at *ε* = 0.25 and 0.33, indicating a focal spot-dominated condition. As the ellipticity increases to *ε* = 0.5, the PVB becomes more prominent and reaches an intensity comparable to that of the central focal spot. This trend is consistent with the polarization decomposition described above; increasing the ellipticity enhances the relative RCP component, thereby strengthening the PVB channel. Similar behavior is observed at 632.8 nm, as shown in Figure 4b(iv–vi), demonstrating that the ellipticity-controlled intensity-tuning method is effective at both operating wavelengths. The average intensities of the focal spot *I*_F_ and PVB *I*_PVB_ regions, which are extracted from the normalized total intensity distributions, as functions of ellipticity at 532nm and 632.8 nm are shown in Figure 4c and Figure 4d, respectively. For each ellipticity, the simulated total intensity distribution is normalized to its maximum value. At both wavelengths, *I*_F_ decreases with the increase in ellipticity, whereas *I*_PVB_ increases correspondingly. The two intensities become comparable at an intermediate ellipticity, demonstrating continuous tuning of the relative intensities between the focal spot and PVB. Figure 4e shows relative intensity *R_I_* = *I*_PVB_/*I*_F_ between the PVB and focal spot as a function of ellipticity. The solid lines in Figure 4e are the fitting curves obtained from Equation (13) with *C*_532_ = 0.12 and *C*_632.8_ = 0.18, which agree well with the simulation results.

The above results demonstrate that without reconfiguration of the metasurface, the relative contributions between the generated focal spot and PVB can be continuously tuned by changing the ellipticity of the incident light. This tunability originates from the intrinsic polarization-dependent weighting of the two geometric-phase-encoded channels. By combining this intrinsic property with wavelength-selective ability and polarization-dependent wavefront control, the proposed metasurface enables discrete optical mode selection and continuous tuning of the relative modal contributions within a single device.

### 3.3. Expanding Optical Functionality with Additional Design Degrees of Freedom

**Figure 5:**
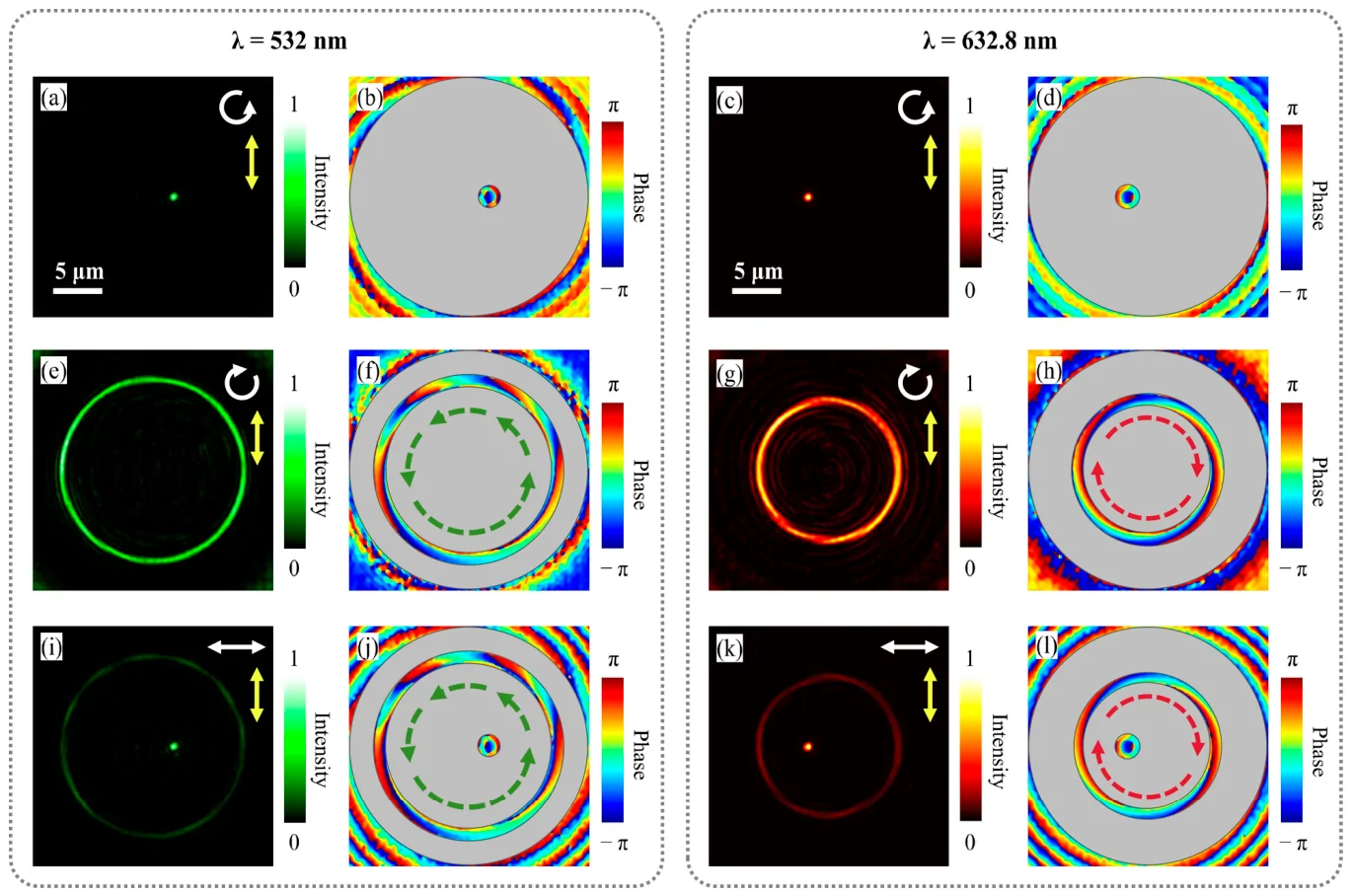
Extended functionality of the metasurface by additional design degrees of freedom. Under LCP light illumination, off-axis focal spots appear at (**a**,**b**) 532 nm and (**c**,**d**) 632.8 nm. Under RCP light illumination, (**e**,**f**) a PVB with non-uniform phase distribution and a topological charge *l* = +4 is generated at 532 nm, while (**g**,**h**) a PVB with a reduced ring radius and a topological charge *l* = −2 is generated at 632.8 nm. Under HLP light illumination, the superposition of LCP and RCP channels produces hybrid optical fields composed of off-axis focal spots and PVBs at (**i**,**j**) 532 nm and (**k**,**l**) 632.8 nm.

To demonstrate the versatility of the design methodology, the design parameters are expanded to generate off-axis focal spots and PVBs with tailored properties, including laterally shifted focal positions, non-uniform phase profile, and different ring radius. The simulated results are presented in Figure 5. Under LCP illumination, off-axis focal spots are clearly observed at the designed positions for both wavelengths, as shown in Figure 5a–d. The generated focal spots are located at (2 μm, 0) and (−2 μm, 0) at 532 nm and 632.8 nm, respectively. The corresponding phase distributions further confirm the successful off-axis focusing at both wavelengths. Under RCP illumination, as shown in Figure 5e,f, a PVB with a ring radius of 9 μm and a topological charge of *l* = +4 is generated at 532 nm. Notably, the phase distribution is designed to be non-uniform. A phase variation of 6π is accumulated over the azimuthal range of 0° to 180°, while a phase variation of 2π occurs from 180° to 360°. The asymmetric phase distributions can be clearly observed in the phase map, demonstrating the capability of the holographic metasurface to encode complex, non-uniform phase distributions beyond conventional vortex beams. At 632.8 nm, as shown in Figure 5g,h, a PVB with a ring radius of 7 μm and a topological charge of *l* = −2 is successfully generated, confirming that the ring radius can be adjusted through the holographic design. Under HLP illumination, hybrid optical fields composed of off-axis focal spots and PVBs are observed at both wavelengths, as shown in Figure 5i–l.

These results demonstrate that the proposed design method is not limited to a specific set of parameters and can be extended to different focal positions, ring radii, topological charges and phase distributions. The flexibility highlights the potential of the proposed metasurface design method for a wide range of applications requiring specific optical-field generation.

## 4. Conclusions

In conclusion, we designed a multifunctional dielectric metasurface that enables on-demand generation of focal spots and PVBs by wavelength- and polarization-control. By designing wavelength-selective Si nanopillars and using the P-B phase method to encode the calculated holographic phase distributions, the metasurface successfully generates focal spots under LCP illumination and PVBs under RCP illumination at both 532 nm and 632.8 nm. The generated PVBs exhibit a common ring radius for different topological charges, with high relative mode purities of 79.7% for *l* = +4 and 97.7% for *l* = −2. Under HLP illumination, the coherent superposition of the LCP and RCP channels generates a hybrid optical field composed of a focal spot and a PVB. The relative contributions between the focal spot and PVB can be continuously tuned by changing the ellipticity of the incident light, enabling continuously tunable hybrid compositions without the metasurface reconfiguration. The design flexibility is further demonstrated by generating off-axis focal spots, PVBs with non-uniform phase distribution and different ring radius. These results demonstrate that the proposed holographic metasurface provides an effective platform for multifunctional, reconfiguration-free, on-demand optical-field generation, with potential applications in optical trapping, quantum information processing, and integrated photonic devices.

## Figures and Tables

**Figure A1:**
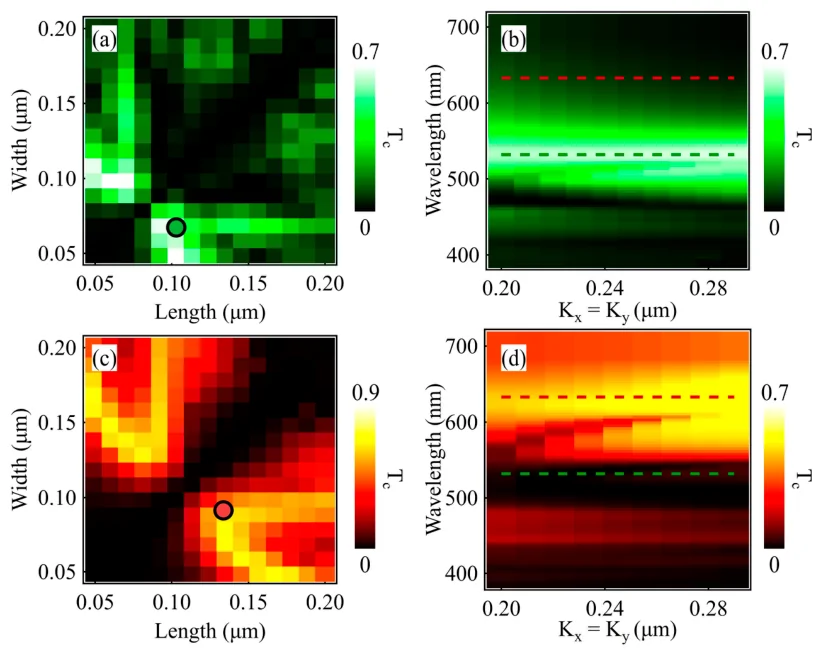
Optimization of the wavelength-selective Si nanopillars. Cross-polarized transmittance *T_c_* as a function of the nanopillar length *L* and width *W* at a fixed period of 0.29 μm and height of 0.41 μm under RCP illumination at (**a**) 532 nm and (**c**) 632.8 nm. The green and red circles mark the candidate geometries with *T_c_* > 0.5 at the target wavelength and *T_c_* < 0.1 at the non-target wavelength. *T_c_* as a function of the incident wavelength and the lattice period for the candidate geometries marked by (**b**) the green circle and (**d**) the red circle. The green and red dashed lines indicate the wavelengths of 532 nm and 632.8 nm, respectively.

**Figure A2:**
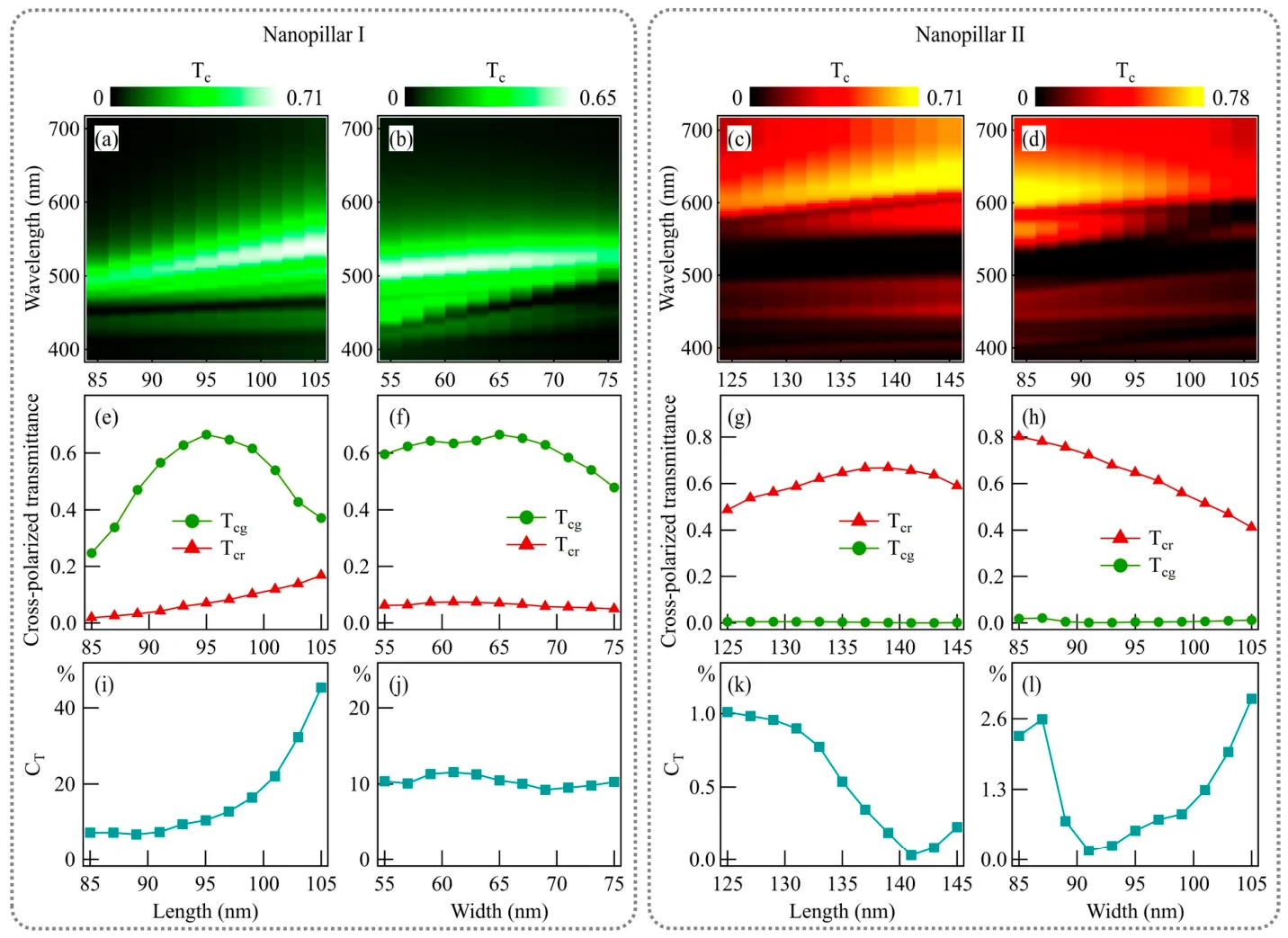
Numerical dimension tolerance analysis of the two Si nanopillars. (**a**–**d**) Wavelength selectivity as functions of lengths and widths of nanopillars. (**e**–**h**) Cross-polarized transmittance at the target and non-target wavelengths as functions of lengths and widths of nanopillars. (**i**–**l**) Corresponding crosstalk ratios as functions of lengths and widths of nanopillars. In each parameter sweep, one geometrical dimension is varied while the other is kept fixed at its desired value.

## Data Availability

The data that support the findings of this study are available from the corresponding author upon reasonable request.

## Appendix A. Parameter Sweep of Wavelength-Selective Nanopillars

The wavelength-selective nanopillar geometries are determined by a systematic numerical parameter sweep. The optimization aims to achieve high cross-polarized transmittance *T_c_* at the target wavelength while suppressing the response at the other operating wavelength. The optimization performs in three steps, including initial geometry screening, lattice-period selection, and fine geometry optimization.

In the initial step, the lattice period is constrained by the spatial-sampling requirements of the designed metasurface. The inner focusing lens has a designed NA of 0.58, while the PVB is generated in an annular region extending from 5 μm to 15 μm. To establish a conservative spatial-sampling constraint for PVB region, we use the maximum effective numerical aperture 0.91, which occurs at the outer radius. To satisfy the Nyquist sampling requirement *K* < λ/2NA at both operating wavelengths and both functional regions, the lattice period should be smaller than 0.292 μm. Therefore, a period of 0.29 μm is chosen for the coarse sweep, which satisfies this bound and provides sufficient sampling for both the focusing and PVB regions. The height is fixed at 0.41 μm., which is within the typical range for Si in the visible wavelength and avoids an excessively large width-to-height ratio. With the period and height fixed, the length *L* and width *W* are swept from 50 nm to 200 nm in steps of 10 nm, and *T_c_* is calculated at both 532 nm and 632.8 nm. The corresponding results are shown in Figure A1a,c. Two candidate geometries 100 nm × 90 nm and 140 nm × 90 nm are identified with *T_c_* > 0.5 at the target wavelength and *T_c_* < 0.1 at the non-target wavelength, as marked by the green and red circles in Figure A1a and Figure A1c, respectively. Using the two candidate geometries, the lattice period is then varied to examine the evolution of *T_c_* with the incident wavelength and the period, as shown in Figure A1b,d. A period of 230 nm is selected to provide relatively narrow spectral responses for the candidate geometries and is more fabrication-friendly. With the period fixed at 230 nm, a refined sweep is performed around each candidate geometry, with length and width extended by 10 nm in each direction and a step of 5 nm. The final dimensions are selected and the *T*_c_ spectra peak is located at the target wavelength with *T*_c_ > 0.6 while *T*_c_ at the non-target wavelength remains below 0.1. This procedure leads to *L_1_* = 95 nm and *W_1_* = 65 nm for 532 nm and *L_2_* = 135 nm and *W_2_* = 95 nm for 632.8 nm. The corresponding spectra are shown in Figure 2b of the main text.

## Appendix B. Dimension Tolerance Analysis

To assess the wavelength selectivity and crosstalk ratios of the proposed metasurface to common fabrication-induced dimensional deviations, we investigate the dimensional tolerances of the two nanopillars. Because the optical response of the metasurface relates to the local electromagnetic behavior of individual nanopillars, unit-cell analysis provides a useful assessment of the device’s sensitivity to fabrication errors. In each parameter sweep, the wavelength selectivity and cross-polarized transmittance are obtained by independently varying the length or width of each nanopillar around its designed value while keeping the other geometrical parameters fixed. The results in Figure A2 illustrate the dimension tolerance responses of the two nanopillars on their lengths and widths.

As shown in Figure A2a–d, increasing the length or width of the nanopillars leads to a redshift of the spectral distribution of the cross-polarized transmittance *T_c_*, indicating that dimensional deviations can change the wavelength selectivity of the two designed nanopillars. This behavior may be attributed to the enhancement of the effective refractive index and the resulting phase accumulation. To quantitatively evaluate the impact of fabrication errors on nanopillar performance, the cross-polarized transmittance at the operating wavelengths of 532 nm and 632.8 nm are simulated as functions of lengths and widths of nanopillars, as presented in Figure A2e–h. The crosstalk ratios *C_T_* are obtained from these transmittance data using Equation (10), as plotted in Figure A2i–l. A critical observation is that Nanopillar I is significantly sensitive to the length variations, and the crosstalk ratio increases with length. As shown in Figure A2e, a ±10 nm length deviation from 95 nm leads to a significant decrease in *T_cg_* and an increase in *T_cr_*, which causes the calculated *C_T_* in Figure A2i to rise rapidly as the length exceeds 100 nm. In contrast, the width variation exhibits a much weaker influence, as presented in Figure A2f,j. Although *T_cg_* fluctuates slightly with width, *T_cr_* remains nearly constant, and the crosstalk ratio *C_T_* stays around 10% from 55 nm to 75 nm. Therefore, for Nanopillar I, the length dimension should be controlled within ±5 nm, whereas the width can tolerate ±10 nm variations. For Nanopillar II, it exhibits an excellent tolerance to dimensional deviations. As shown in Figure A2g,h, *T_cr_* remains above 50% across the entire ±10 nm length variation, and decreases from 80% to 40% with width increase from 85 nm to 105 nm, while *T_cg_* stays negligibly low. In both cases, *C_T_* in Figure A2k,l remains within 3% throughout the dimension variation. This robustness can be attributed to the relatively lower aspect ratio and the broader resonance band of Nanopillar II. These findings provide practical guidelines for fabrication. For Nanopillar I, length accuracy is the dominant factor, whereas for Nanopillar II, both dimensions can be relaxed to ±10 nm without sacrificing the polarization filtering capability.
